# Confirmation of the Cardioprotective Effect of MitoGamide in the Diabetic Heart

**DOI:** 10.1007/s10557-020-07086-7

**Published:** 2020-09-26

**Authors:** Min Park, Takanori Nishimura, Carlos D. Baeza-Garza, Stuart T. Caldwell, Pamela Boon Li Pun, Hiran A. Prag, Tim Young, Olga Sauchanka, Angela Logan, Marleen Forkink, Anja V. Gruszczyk, Tracy A. Prime, Sabine Arndt, Alba Naudi, Reinald Pamplona, Melinda T. Coughlan, Mitchel Tate, Rebecca H. Ritchie, Federico Caicci, Nina Kaludercic, Fabio Di Lisa, Robin A. J. Smith, Richard C. Hartley, Michael P. Murphy, Thomas Krieg

**Affiliations:** 1 Department of Medicine, University of Cambridge, Cambridge, UK; 2 MRC Mitochondrial Biology Unit, University of Cambridge, Cambridge, UK; 3 Takeda Pharmaceutical Ltd, Tokyo, Japan; 4 WestCHEM School of Chemistry, University of Glasgow, Glasgow, UK; 5 Department Of Experimental Medicine, University of Lleida, Lleida Institute for Biomedical Research, Lleida, Spain; 6 Department of Diabetes, Monash University, Melbourne, Australia; 7 Baker Heart and Diabetes Institute, Melbourne, Australia; 8 Department of Biology, University of Padova, Padua, Italy; 9 Neuroscience Institute, National Research Council of Italy (CNR), Pisa, Italy; 10 Department of Biomedical Sciences, University of Padova, Padua, Italy; 11 Department of Chemistry, University of Otago, Otago, New Zealand

**Keywords:** Diabetes, Heart failure with preserved ejection fraction (HFpEF), Akita mice, Advanced glycation endproducts (AGE), Mitochondria

## Abstract

**Purpose:**

HFpEF (heart failure with preserved ejection fraction) is a major consequence of diabetic cardiomyopathy with no effective treatments. Here, we have characterized Akita mice as a preclinical model of HFpEF and used it to confirm the therapeutic efficacy of the mitochondria-targeted dicarbonyl scavenger, MitoGamide.

**Methods and Results:**

A longitudinal echocardiographic analysis confirmed that Akita mice develop diastolic dysfunction with reduced E peak velocity, E/A ratio and extended isovolumetric relaxation time (IVRT), while the systolic function remains comparable with wild-type mice. The myocardium of Akita mice had a decreased ATP/ADP ratio, elevated mitochondrial oxidative stress and increased organelle density, compared with that of wild-type mice. MitoGamide, a mitochondria-targeted 1,2-dicarbonyl scavenger, exhibited good stability in vivo, uptake into cells and mitochondria and reactivity with dicarbonyls. Treatment of Akita mice with MitoGamide for 12 weeks significantly improved the E/A ratio compared with the vehicle-treated group.

**Conclusion:**

Our work confirms that the Akita mouse model of diabetes replicates key clinical features of diabetic HFpEF, including cardiac and mitochondrial dysfunction. Furthermore, in this independent study, MitoGamide treatment improved diastolic function in Akita mice.

**Electronic supplementary material:**

The online version of this article (10.1007/s10557-020-07086-7) contains supplementary material, which is available to authorized users.

## Introduction

Heart failure with preserved ejection fraction (HFpEF) is an early cardiac manifestation found in young diabetic individuals, whereas heart failure with reduced ejection fraction (HFrEF) develops predominantly in older age groups [1]. Despite the prevalence of HFpEF among diabetic patients, the underlying pathophysiological mechanisms are unclear, limiting the development of rational therapies. Indeed, currently available anti-diabetic drugs are of limited efficacy against both HFpEF and HFrEF, and the associated increase in mortality [2]. Hence, there is a clear unmet need to more effectively target cardiac damage in diabetes to protect against heart failure. A major impediment to developing drugs for HFpEF is the lack of preclinical animal models that closely replicate the human pathology.

The glucose elevation associated with diabetes increases formation of reactive 1,2-dicarbonyls, such as methylglyoxal and glyoxal, with elevated levels in diabetic patients [3]. Methylglyoxal and glyoxal are a major cause of the accumulation of protein glycation and of advanced glycation endproducts (AGEs) in diabetes [4]. These modifications are thought to disrupt protein function and are implicated in the pathogenesis of diabetes and in its related cardiovascular complications [4, 5]. A number of clinical studies have linked elevated AGEs in the plasma proteins and skin collagen of diabetic patients to the risk of vascular complications [6, 7]. In preclinical studies, the pathological role of dicarbonyl glycation has been demonstrated using pharmacological inhibitors, or by altering expression of the dicarbonyl detoxifying enzyme, glyoxalase I (*Glo I*) [8–10].

**Fig. 1:**
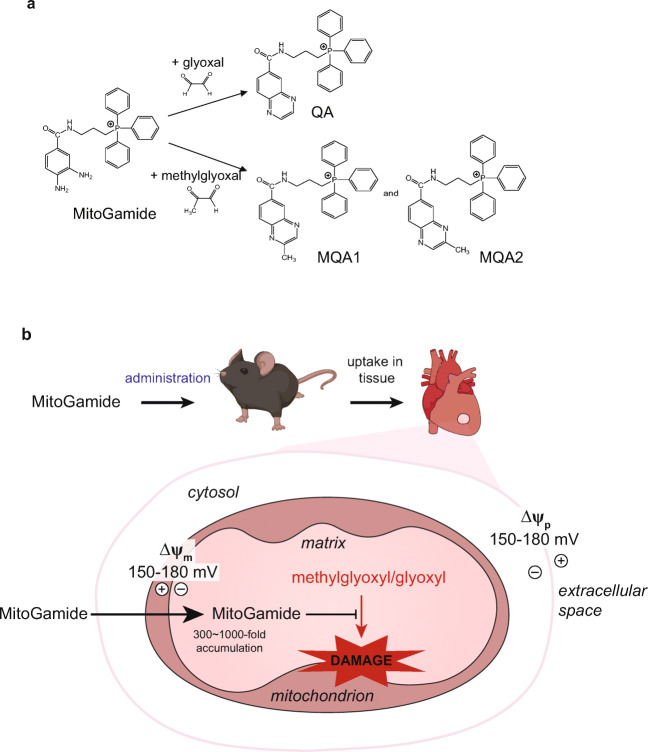
Activity of MitoGamide. **a** The reactions of MitoGamide with glyoxal to form quinoxaline amide (QA) and methylglyoxal to form methylquinoxaline amide (MQA: note that there are two regioisomers of MQA). **b** Schematic showing the effect of elevated glucose on mitochondrial dysfunction leading to diabetic cardiomyopathy and its prevention by MitoGamide

As mitochondrial damage due to reactive 1,2-dicarbonyls contributes to diabetic heart failure, sequestering 1,2-dicarbonyls within mitochondria before they cause damage suggests itself as a potential therapy. Previously, we developed a probe called MitoG to assess the mitochondrial levels of methylglyoxal and glyoxal [11]. MitoG incorporates a triphenylphosphonium (TPP) moiety that drives the compound’s selective accumulation within mitochondria due to the membrane potential [11, 12]. MitoG also contains an *o*-phenylenediamine moiety that reacts selectively with methylglyoxal and glyoxal to form stable quinoxaline products that can be quantified by LC-MS/MS [11]. This approach enabled mitochondrial dicarbonyl levels to be assessed in vivo. These findings suggested that a mitochondria-targeted molecule that sequestered 1,2-dicarbonyls might have therapeutic potential by decreasing mitochondrial damage in diabetic cardiomyopathy. However, because MitoG is susceptible to oxidation, it was too short lived to be effective as a therapy. Therefore, we developed a longer-lived analogue, MitoGamide (Fig. 1a). This was done by removing electron density from the aryl diamine by using an electron withdrawing amide linkage (Fig. 1a), rather than an electron-donating ether link as was used in MitoG. MitoGamide should react with and sequester glyoxal and methylglyoxal to form the inactive products methylquinoxaline amide (MQA) and quinoxaline amide (QA) (Fig. 1a). Furthermore, its enhanced stability is intended to enable MitoGamide to accumulate in cardiac mitochondria, sequester reactive 1,2-dicarbonyls and thereby decrease mitochondrial damage and ameliorate diabetic cardiomyopathy (Fig. 1b). In a preliminary study, MitoGamide was protective against diabetic cardiomyopathy in an Akita mouse model of experimental diabetes [13].

Here, we set out to confirm and extend the results of MitoGamide in an independent investigation, an important requirement for the robust validation of cardioprotective drugs. As preclinical models to assess therapies for HFpEF are not well developed, we first characterized the cardiomyopathy in the Akita mouse model of diabetes. These mice arose spontaneously from a C57BL6/6NSlc background and have minimal insulin secretion leading to chronically elevated plasma glucose [14, 15]. Here, we show that Akita mice develop diabetic pathologies, including HFpEF, and hence can be used to assess potential therapies. When we treated Akita mice with MitoGamide, we found that it decreased the development of diastolic dysfunction, raising the prospect that mitochondria-targeted therapies may help in treating diabetic cardiomyopathy.

## Methods

### Animal Use and Echocardiography

Wild-type C57BL/6 J (Charles River Laboratories, UK) and heterozygous *Ins2*^*Akita*^ (The Jackson Laboratory, USA) male mice were used for experiments. *Ins2*^*Akita*^, hereafter called Akita, carry a single nucleotide substitution in the insulin 2 gene (*Ins2*). This mutation results in misfolding of proinsulin 2 and eventually leads to ER stress and pancreatic β cell failure and ultimately to severe hyperglycaemia. At 5 weeks of age, Akita mice developed hyperglycaemia with non-fasting blood glucose levels higher than 25 mM. For chronic MitoGamide treatment, 6-week-old mice were subjected to daily oral administration via gavage of MitoGamide (10 mg/kg) or vehicle (sterile water) for 12 weeks. Blood glucose and HbA1c levels (service provided by Core Biochemical Assay Laboratory, Cambridge University Hospital) were monitored during the treatment period. Cardiac function and morphology were evaluated using echocardiography (Vevo 3100, VisualSonics). The parasternal long-axis view (B mode), the short-axis view (M mode) and blood flow velocity (PW mode) at mitral valve were obtained, and measurements of cardiac structure and function were accessed using Vevo Lab (VisualSonics) in a completely blinded manner.

### MitoP/B: Measurement of Mitochondrial Oxidative Stress In Vivo

As previously described [16], the mitochondria-targeted probe MitoB reacts with hydrogen peroxide and is converted to MitoP. The ratio of MitoP/MitoB is used as a quantitative measurement indicating mitochondrial H_2_O_2_ levels. MitoB (1 μmol/kg) was injected intravenously via tail vein. After 3 h, tissues were collected and snap frozen in liquid N_2_, subsequently stored at − 80 °C until LC-MS analysis.

### ATP/ADP Assay

See supplementary information.

### Transmission Electron Microscopy Image Analysis

See supplementary information.

### Chemical Synthesis

See supplementary information.

### Quantification of MitoGamide, MQA and QA Using LC-MS/MS

See supplementary information.

### Cellular Uptake of MitoGamide

C2C12 myoblasts were maintained in DMEM supplemented with 10% foetal bovine serum and 1% penicillin/streptomycin at 37 °C with 5% CO_2_ in a humidified incubator. Two days prior to experiment, cells were seeded in a 6-well plate with normal culture medium. When cells reached 60–70% confluency, the MitoGamide at various concentrations (0, 10, 50 and 100 nM) was added to each well. After 1 h or 24 h incubation time, cells were collected using cell scrapers after rinsing with PBS. Cell pellets were collected by centrifugation at 16000×*g* for 3 min. The supernatant was discarded, and cell pellets were stored at − 80 °C until LC-MS/MS analysis (see supplementary information).

### Mitochondrial Uptake of MitoGamide Using RP-HPLC

Freshly isolated rat heart mitochondria (1-mg protein/ml) were incubated in 1-ml pre-warmed KCl buffer supplemented with rotenone (4 μg/ml), internal standard propyl triphenylphosphonium (PTPP; 5 μM) and MitoGamide (5 μM) in 2-ml microtubes. Respiration was initiated by the addition of succinate (5 mM) with or without FCCP (500 nM). After 5 min of incubation in a shaking water bath at 37 °C, mitochondria were pelleted by centrifugation (7500×*g* for 10 min at 4 °C). Supernatants (750 μl) were removed and stored at − 80 °C in microtubes until analysis. Mitochondrial pellets were extracted in 250-μl buffer B (acetonitrile + 0.1% trifluoroacetic acid (TFA)) followed by centrifugation (7500×*g* for 5 min). All samples were diluted to 25% acetonitrile with buffer A (water + 0.1% TFA) and separated and analysed by RP-HPLC.

### Cell Viability Assay

See supplementary information.

### Western Blot Analysis

See supplementary information.

### mRNA Quantification by qPCR

See supplementary information.

### Mitochondrial DNA Damage

See supplementary information.

### GC-MS Analysis of Protein Glycation Markers

Markers of protein glycoxidation (N^ε^-(carboxyethyl)-lysine [CEL], N^ε^-(carboxymethyl)-lysine [CML], and S-(carboxymethyl)-cysteine [CMC]) were determined as trifluoroacetic acid methyl esters (TFAME) derivatives in acid hydrolyzed delipidated and reduced tissue/mitochondrial protein samples by GC/MS using a HP6890 Series II gas chromatograph (Agilent) with a MSD5973A Series detector and a 7683 Series automatic injector, a HP-5MS column (30 m × 0.25 mm × 0.25 μm), and the described temperature program [17, 18]. Quantification was performed by internal and external standardization using standard curves constructed from mixtures of deuterated and non-deuterated standards. Analyses were carried out by selected ion-monitoring GC/MS (SIM-GC/MS). The ions used were as follows: lysine and [^2^H_8_]lysine, m/z 180 and 187, respectively; CEL and [^2^H_4_]CEL, m/z 379 and 383, respectively; CML and [^2^H_2_]CML, m/z 392 and 394, respectively; and CMC and [^13^C_3_-^15^N]CMC, m/z 271 and 273, respectively. The amounts of product were expressed as μmoles of CEL, CML or CMC per mole of lysine.

### Statistics

Data are presented as means ± SEM. Comparisons of a single variable in ≥ 2 groups were analysed by Student’s *t* test or one-way ANOVA followed by Tukey’s multiple comparison tests (GraphPad Prism). Values of *p* < 0.05 were considered significant.

## Results

### Akita Mice as a Preclinical Model of Diabetic HFpEF

**Fig. 2:**
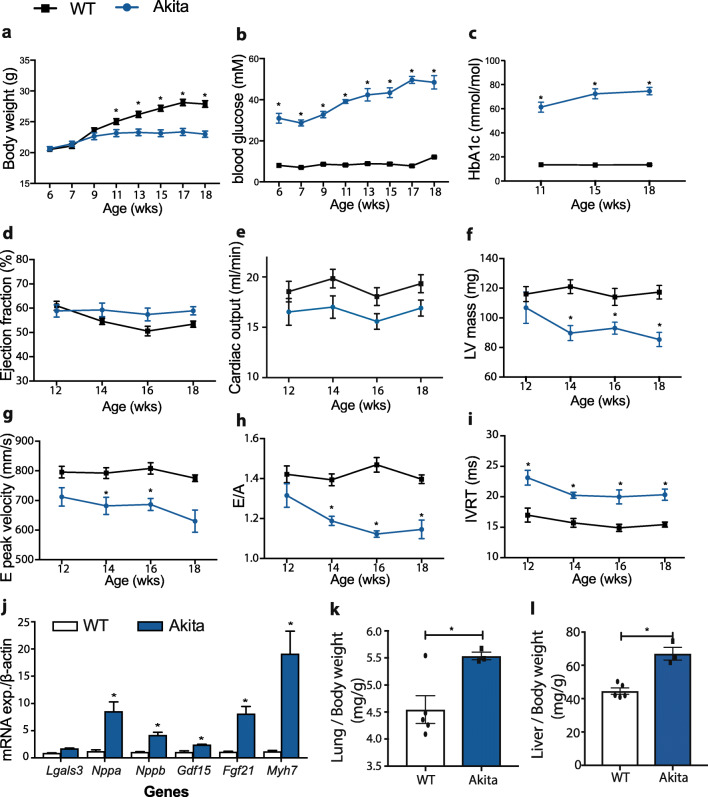
Comparison between wild-type and Akita mice in **a** body weight (*n* = 11–15), **b** non-fasting blood glucose levels (*n* = 11–15) and **c** plasma HbA1c levels (*n* = 4–6). Longitudinal echocardiographic analysis of Akita and wild-type hearts was used to measure left ventricular (LV) ejection fraction (EF) (**d**), cardiac output (CO) (**e**), LV mass (**f**), E peak (**g**), E/A ratio (**h**) and isovolumetric relaxation time (IVRT) (**i**)**.** Values are expressed as mean ± SEM (*n* = 11–15). (**j**) shows mRNA expression of biomarkers of heart failure in heart tissues (*n* = 4–6); *Lgals3* galectin-3, *Nppa* natriuretic peptide-A, *Nppb* natriuretic peptide-B, *Gdf15* growth differentiation factor 15, *Fgf21* fibroblast growth factor 21, *Myh7* myosin heavy chain 7. (**k-l**) show the difference between wild-type and Akita mice in lung and liver weights (mg), normalized by body weight (g). Statistical significance has been tested with Student’s *t* test

To validate the protective effects of MitoGamide described previously, we first independently confirmed the phenotype of Akita mice. Body weight, blood glucose and HbA1c in Akita mice were monitored from 6 weeks of age and compared with C57BL6/J wild-type mice (Fig. 2). In agreement with previous reports [19, 20], Akita mice were smaller and developed severe hyperglycaemia when compared with wild-type mice (Fig. 2a–c). Cardiac functional and structural changes were monitored by echocardiography every 2 weeks from 12 weeks of age (Fig. 2d–i). At 18 weeks of age, Akita mice developed diastolic dysfunction, evidenced by a significantly reduced E peak velocity, reduced E/A ratio (< 1.15) and extended isovolumetric relaxation time (> 20.0 ms), measured via mitral valve blood flow (Fig. 2g–i ). Even so, systolic function, represented by ejection fraction (EF) and cardiac output (CO), was comparable between Akita and wild-type mice (Fig. 2d–e). Finally, analysis of mRNA expression in hearts from Akita mice showed an upregulation in expression of a number of genes associated with heart failure [21], including *Nppa, Nppb, Gdf15, Fgf21* and *Myh7* (Fig. 2j). Furthermore, Akita mice showed increased relative lung and liver weights, which has to be seen in light of the reduced overall weight but might indicate congestive HF-associated peripheral oedema in Akita mice (Fig. 2k–l). There was no difference in inflammatory markers between wild-type and Akita mice (Fig. S3). The combination of diastolic dysfunction with preserved systolic function replicates some of the clinical features of early-stage diabetic cardiomyopathy in humans [1].

**Fig. 3:**
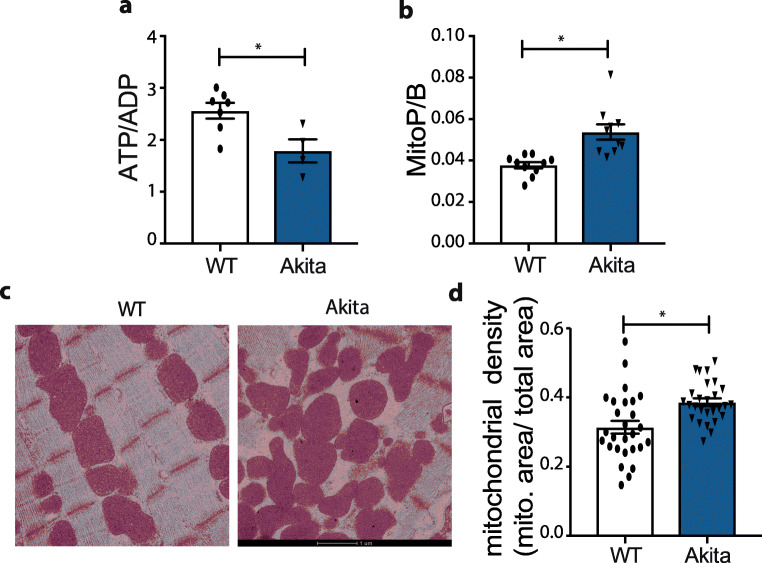
Mitochondrial function in Akita mice. **a** Measurement of ATP/ADP ratio in the myocardial tissue of Akita and wild-type mice (*n* = 4–7). **b** Measurement of mitochondrial hydrogen peroxide levels assessed by measuring MitoP/B ratio using LC-MS/MS (*n* = 10). **c** Representative TEM images of heart tissues from wild-type and Akita heart tissues. **d** Quantification of mitochondrial density in cardiomyocytes accessed by TEM. Values are expressed as mean ± SEM, and statistical significance has been tested with Student’s *t* test

We next analysed mitochondrial function in Akita mice at 16 weeks of age (Fig. 3a–d). The cardiac ATP/ADP ratio of Akita mice was ~ 30% lower than for wild-type mice (Fig. 3a). Mitochondrial H_2_O_2_ levels in vivo, measured using the mitochondria-targeted MitoB probe [16], showed higher hydrogen peroxide levels in the cardiac mitochondria of Akita mice compared with controls (Fig. 3b). Transmission electron micrographs (TEM) of cardiac tissue showed disruption in mitochondrial morphology in Akita mice, compared with wild type (Fig. 3c). Quantification of electron micrographs indicated that the number of mitochondria was not altered (data not shown), but that there was an increase in mitochondrial density (Fig. 3d). We conclude that Akita mice develop heart dysfunction similar to that found in HFpEF patients and that this pathology is associated with mitochondrial dysfunction. Importantly, we also showed that this cardiac phenotype is conserved as these results mirror the previous study, despite being carried out at independent centres.

### In Vitro Properties of MitoGamide

The findings described above are consistent with elevated glucose leading to mitochondrial dysfunction and cardiomyopathy in Akita mice. A plausible pathway contributing to mitochondrial dysfunction is through glycation of mitochondrial proteins by the elevated 1,2-dicarbonyls that arise as a consequence of high glucose levels in the Akita mice. Therefore, we generated a mitochondria-targeted compound, MitoGamide (Fig. S1), that accumulates within mitochondria and sequesters the damaging 1,2-dicarbonyls that contribute to mitochondrial damage and heart dysfunction (Fig. 1b).

MitoGamide was based on MitoG [11], but altered to incorporate an electron withdrawing amide linker between the mitochondria-targeting TPP moiety and the dicarbonyl sequestering phenylenediamine moieties (Fig. S1). This was done to decrease oxidation of the phenylenediamine so as to extend MitoGamide’s lifetime in vivo while still sequestering 1,2-dicarbonyls. The synthesis of MitoGamide and related compounds are shown in Fig. S1.

**Fig. 4:**
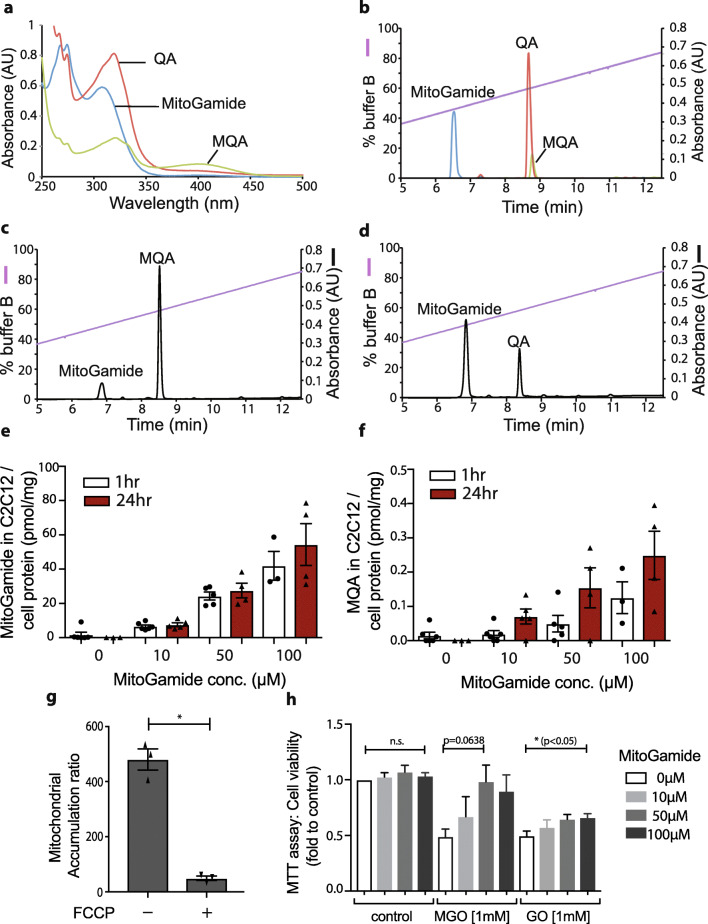
In vitro characterization of MitoGamide. **a** UV/Vis scanning spectra of 100 μM MitoGamide, MQA and QA in KCl buffer. **b** RP-HPLC profile of 10-nmol MitoGamide, MQA and QA made up fresh in DMSO. Absorbance was measured at 220 nm. **c,d** Reaction mixtures consisting of 5-mM MitoGamide and 10-mM methylglyoxal or glyoxal in a final volume of 10 μl in KCl buffer A were incubated at 37 °C for 2 h. For RP-HPLC, 1 μl of each mixture was used. **e,f** Dose-dependent cellular uptake level of MitoGamide and time-dependent accumulation of MQA in C2C12 cells, measured by LC-MS/MS (*n* = 3–6). Values are mean ± SEM. **g** MitoGamide accumulation by isolated rat liver mitochondria, measured by HPLC (*n* = 3). **h** Effect of MitoGamide at various doses in C2C12 cell viability accessed by MTT assay when cells were exposed to exogenously added methylglyoxal or glyoxal (1 mM) (*n* = 4). The effects of MitoGamide treatment have been tested by one-way ANOVA

The UV absorption spectrum and the RP-HPLC analysis of MitoGamide and its products from reaction with glyoxal (QA) and methylglyoxal (MQA) are shown in Fig. 4a and b. RP-HPLC analysis of MitoGamide following reaction with methylglyoxal (Fig. 4c) or glyoxal (Fig. 4d) showed that MitoGamide reacted with 1,2-dicarbonyls to form the expected products, MQA and QA, respectively. MitoGamide accumulated ~ 500-fold within energized mitochondria, and this accumulation was prevented by abolishing the mitochondrial membrane potential with the uncoupler FCCP (Fig. 4g). MitoGamide was also taken up into C2C12 myoblasts in a dose-dependent manner (Fig. 4e). The MitoGamide uptake was unchanged from 1 to 24 h, indicating that a stable steady-state distribution was rapidly established and that MitoGamide was stable within cells. Incubation with MitoGamide led to the accumulation of MQA, the reaction product of MitoGamide with methylglyoxal (Fig. 4f), indicating that MitoGamide reacts with the methylglyoxal generated spontaneously during glucose metabolism. In contrast, there was a negligible accumulation of QA, the product from the reaction of MitoGamide with glyoxal (data not shown). Treating C2C12 cells with exogenous methylglyoxal or glyoxal led to ~ 50% loss of viability (Fig. 4h). MitoGamide showed a dose-dependent protection against methylglyoxal toxicity (Fig. 4h). MitoGamide was less protective against glyoxal, probably due to the greater reactivity of methylglyoxal with MitoGamide. This arises because methylglyoxal is mostly present as the monohydrate that requires a single dehydration to generate the reactive dicarbonyl, whereas glyoxal is a dihydrate making it less reactive.

Together, these data indicate that MitoGamide is taken up by mitochondria and cells, where it is stable and reacts with methylglyoxal, thereby protecting against the damage caused by 1,2-dicarbonyls.

### Glycation Endproducts in Akita Mice

**Fig. 5:**
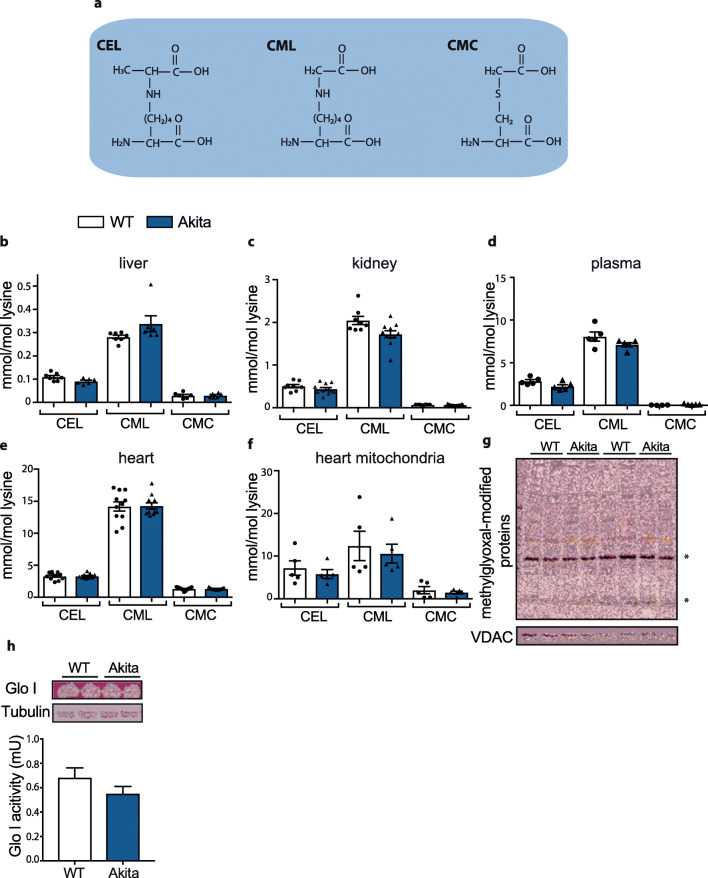
**a** Structures of CEL, CML and CMC. **b–e** Tissue accumulation of methylglyoxal or glyoxal-mediated AGEs, CEL, CML and CMC were accessed using GC-MS in wild-type and Akita mice (12–15 weeks old) (*n* = 8–11). **f,g** Data representing the AGE levels in mitochondrial fractions of heart tissues from wild-type or Akita mice, assessed by **f** GC-MS (*n* = 5) and **g** Western blot analysis. **h** Representative Western blot comparing glyoxalase I protein expression and activity between wild-type and Akita (*n* = 4)

Reactive dicarbonyls such as methylglyoxal and glyoxal are thought to react primarily with lysine, arginine and cysteine residues in proteins [17, 18, 22]. The resulting adducts include Nε-(carboxymethyl)-lysine (CML), Nε-(carboxyethyl)-lysine (CEL) and *S*-(carboxymethyl)-cysteine (CMC) [16, 17] (Fig. 5a). To extend our knowledge on the Akita mouse model and its phenotype, we measured these adducts by GC-MS in tissues from wild-type and Akita mice (Fig. 5b–f). Unexpectedly, the liver, kidney, plasma and heart from Akita mice did not show increased levels of these adducts compared with wild-type mice (Fig. 5b–e). To see whether these adducts might be enriched in the mitochondria, we assessed heart mitochondria by GC-MS (Fig. 5f) and by Western blotting using an antibody selective for the methylglyoxal-modified arginine adduct argpyrimidine (Fig. 5g). However, there was again no difference in adduct formation between wild-type and Akita mice.

We next considered whether the lack of increased protein glycation in Akita mice, despite elevated glucose, was due to a compensatory upregulation of defences against protein glycation. The major enzyme responsible for methylglyoxal and glyoxal detoxification is glyoxalase I (*Glo I*) [5, 8, 23]. However, Akita mice did not upregulate *Glo I* expression or activity (Fig. 5h).

### MitoGamide Distribution In Vivo

**Fig. 6:**
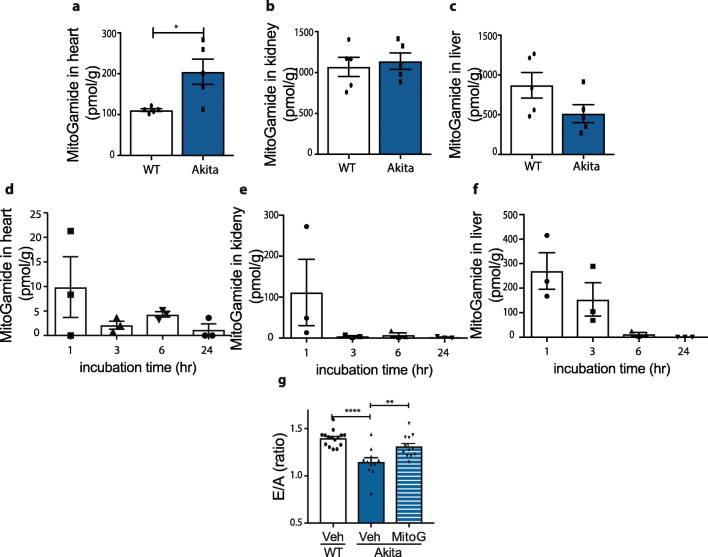
Tissue distribution and in vivo effect of MitoGamide in mice. **a–c** MitoGamide level was measured in tissues, heart, kidney and liver, collected 4-h post i.v. injection (100-nmol/mouse, or equivalent to 2 mg/kg for mice weighing 25–30 g) from both wild-type and Akita mice (*n* = 5). The difference between wild-type and Akita groups has been tested with Student’s *t* test. **d–f** MitoGamide (10 mg/kg, approximately 500 nmol/mouse) was given to mice by oral gavage; then its contents in heart, liver and kidney were measured by LC-MS/MS (*n* = 3). Values are mean ± SEM. Effect of MitoGamide on heart function. **g** The effect of 12 week long MitoGamide treatment (10 mg/kg daily oral gavage) on E/A ratio, accessed by echocardiography (*n* = 11–15). **h** Representative Western blot and the quantification of LC3II expression normalized by LC3I in mitochondrial fractions of heart tissues collected after the 12 weeks treatment (*n* = 3–6). Values are mean ± SEM. Statistical significance has been tested by One-way ANOVA

To see if MitoGamide could be effective as a potential therapeutic, we next assessed its distribution to target tissues in vivo. To do this, we first developed an LC-MS/MS assay for MitoGamide, MQA and QA (Fig. S2) that enabled us to measure their levels in tissues. We assessed the tissue distribution of MitoGamide 4 h after intravenous injection (Fig. 6a–c), which showed a significant MitoGamide uptake into the heart. Interestingly, there were higher levels of MitoGamide in the hearts of Akita mice compared with wild-type mice, possibly due to the increased mitochondrial density in the cardiomyocytes of Akita mice, as reported previously [19] and confirmed by our own TEM analysis (Fig. 3c). Significant amounts of MitoGamide were also detected in the kidney (Fig. 6b) and liver (Fig. 6c) of wild-type and Akita mice. Both MQA and QA were detected in spot urine samples from both wild-type and Akita mice following MitoGamide injection (Fig. S4). Thus, MitoGamide is taken up from the circulation into tissues and there reacts with methylglyoxal and glyoxal. As intravenous injection is not feasible for a clinically relevant long-term MitoGamide administration, we next assessed tissue distribution following administering MitoGamide by oral administration via intragastric gavage (Fig. 6d–f). This showed that there were reasonable amounts of MitoGamide present in the heart (Fig. 6d) and liver (Fig. 6f) for several hours after gavage, while in the kidney, MitoGamide was cleared relatively rapidly (Fig. 6e). Therefore, MitoGamide is taken up into the heart, is reasonably stable and reacts with methylglyoxal and glyoxal in vivo.

### MitoGamide Protects Against Cardiac Dysfunction in Akita Mice

To confirm whether MitoGamide could protect against diabetic cardiomyopathy, we treated Akita mice with MitoGamide daily for 12 weeks by oral gavage and assessed a range of markers of heart function by echocardiography (see Supplementary Table S1). MitoGamide treatment significantly improved the E/A ratio (Fig. 6g). Importantly, the E/A ratio is a key indicator of diastolic dysfunction in the development of diabetes-induced HFpEF in patients [24]. This suggests that MitoGamide counteracts some of the damaging consequences of diabetes that lead to diastolic cardiac dysfunction. MitoGamide did not change body weight, blood glucose or HbA1c in either Akita or wild-type mice (data not shown). This protection against cardiac dysfunction was the same as that seen in the previous study, validating the effectiveness of MitoGamide [13] and following recent guidelines [25].

MitoGamide can intercept reactive 1,2-dicarbonyls and thereby prevent damage (Fig. 4h). However, as there was no increase in markers of protein glycation in the Akita mice compared with controls, it was not possible to assess whether this was how MitoGamide protected against the cardiac dysfunction. Therefore, we assessed the effect of MitoGamide on other indicators of mitochondrial function (Fig. S5a–e). However, we found that MitoGamide did not act by preventing deleterious mitochondrial changes in Akita mice, including ATP/ADP ratio (Fig. S5a), mitochondrial ROS levels (Fig. S5b), expression of genes associated with cardiac dysfunction (Fig. S5c), nor the increase in mitochondrial density (Fig. S5d). In addition, MitoGamide did not affect the levels of mitochondrial DNA damage in Akita mice (Fig. S5e). However, there was an increase in mitochondrial levels of LC3B II and Pink1 in the hearts of Akita mice compared with wild type (Fig. S6a–d), suggesting that Akita mice might upregulate autophagy to adapt to the metabolic changes in the tissue. MitoGamide treatment only reduced LC3B II, while others stayed unaffected; hence, further investigation is needed to fully understand the role of autophagy in the observed protection.

## Discussion

Diabetes is a highly prevalent risk factor in the development of HFpEF, and diabetic HFpEF patients have a marked increase in morbidity and mortality. HFpEF is a complex clinical syndrome which is mainly, but not exclusively, characterized by diastolic cardiac dysfunction, a systemic pro-inflammatory state, and a preserved or only mildly impaired systolic cardiac pump function. HFpEF is now regarded as one of the main forms of chronic heart failure with an increasing prevalence worldwide [26]. In contrast to heart failure with reduced ejection fraction (HFrEF), there are currently no specific therapies for HFpEF. One of the reasons for the failure to develop and test specific HFpEF therapies lies in the lack of suitable preclinical animal models. Here, we have shown that the Akita mouse model of type I diabetes replicates many features of HFpEF, in particular cardiac diastolic dysfunction, making it a potential model to assist in the development of new drugs for this disease. In addition, the development of cardiac phenotypes that replicate those of HFpEF in Akita mice was associated with mitochondrial dysfunction. Mitochondrial dysfunction is considered to be a direct consequence of the systemic pro-inflammatory conditions found in HFpEF, aggravated by a diabetic environment [27, 28]. Although we could not find any changes in the systemic inflammatory state of Akita mice, they showed increased oxidative stress seen by increased mitochondrial H_2_O_2_ production in vivo. Akita mice might develop compensatory mechanisms to counteract chronic inflammation, which will be investigated in future work.

Taken together, in accordance with previous studies [15, 19, 20], the Akita mouse model demonstrated several pathognomonic features of HFpEF, rendering it suitable to test novel treatment options. As the formation of reactive 1,2-dicarbonyls such as glyoxal and methylglyoxal is associated with elevated levels of glucose, we hypothesised that glycation of mitochondrial proteins by these reactive dicarbonyls was a major cause of the mitochondrial dysfunction and cardiac damage in the Akita mice. Hence, we developed a mitochondria-targeted compound, MitoGamide, designed to sequester reactive dicarbonyls. MitoGamide was protective against diastolic dysfunction, one of the major HFpEF components in these mice. Although we hypothesised that MitoGamide protects cells against dicarbonyl damage, it was difficult to assess this mechanism directly in vivo, as there was no elevation of protein glycation in the Akita mice compared with wild-type mice. The lack of accumulation of protein adducts was surprising, given that the elevated glucose in the Akita mice leads to increased amounts of methylglyoxal and glyoxal. This was not due to an upregulation of *glyoxalase I* activity; however, we cannot exclude the possibility that the Akita mice have developed alternative compensatory mechanisms to adapt to the dicarbonyl stress associated with hyperglycaemia. Furthermore, we could not see changes in the expression of key heart failure-associated genes (Fig. S5c), which could indicate that despite MitoGamide improving diastolic dysfunction, there is an ongoing drive from the diabetic state of these mice towards heart failure development. The present study is not providing a clear molecular mechanism of action supporting the cardioprotective effect seen in MitoGamide-treated Akita mice. Diabetic complications in vivo are multi-factorial and often secondary to changes in other AGE-sensitive organs. Therefore, effects on the heart cannot be fully distinguished from effects of MitoGamide in other areas which we have not examined. Indeed, in addition to hyperglycaemia and insulin deficiency, Akita mice are prone to develop retinopathy, nephropathy, atherosclerosis, dyslipidaemia and a higher expression of pro-inflammatory factors [14, 29]. The influence of these changes on the cardiac pathologies and MitoGamide’s protection still warrants further work.

In conclusion, we have demonstrated that the Akita mouse model replicates many cardiac phenotypes observed in diabetes-associated HFpEF at both physiological and molecular levels. Furthermore, the robust protection afforded by MitoGamide, in multiple independent studies, against cardiac myopathy in this model suggests that mitochondria-targeted therapies may be of utility in addressing cardiac dysfunction associated with diabetes. However, the lack of AGE accumulation in Akita mice made it impossible to fully elucidate the in vivo effect of MitoGamide and is also not in line with clinical results showing elevated AGE levels in tissue and plasma of diabetic patients. Moreover, other well-established diabetes mouse models such as *db/db* and high-fat/high-sucrose fed mice have not shown clear evidence of AGE accumulation at the age range which various diabetic phenotypes are detected [30]. This leaves us with a challenge to find a suitable preclinical animal model closely matching with the clinical pathogenesis to test novel therapeutic compounds such as MitoGamide.

## Electronic Supplementary Material

ESM 1 (DOCX 35 kb)

ESM 2 Echocardiogrpahy measurements comparing vehicle-treated wild-type, vehicle-treated Akita, and MitoGamide (10 mg/kg given via daily oral gavage for 12 weeks) treated Akita mice at 18 weeks of age. EF; ejection fraction, FS; fractional shortning, CO; cardiac output, SV; stroke volume, LVEDV; left ventricular end-diastolic volume, LVESV; left ventricular end-systolic volume, LVAW; left ventricular anterior wall thickness, LVPW; left ventricular posterior wall thickness, LV mass; left ventricle mass, AoV; peak aortic valve velocity, MV E; mitral valvle E (early) peak velocity; MV A; mitral valve A (atrial contraction) peak velocity, IVRT; isovolumetric relaxation time, IVCT; isovolumetric contraction time, AET; aortic ejection time, E Decel; E peak deceleration, HR; heart rate. Values are mean ± SEM (*n* = 11–15).Statistical significance has been tested by One-way ANOVA. (PDF 50 kb)

ESM 3 **Supplementary Fig. 1** Chemical syntheses of MitoGamide, MQA and QA. **Supplementary Fig. 2** LC-MS/MS quantification of MitoGamide and its products. **(a)** Typical LC-MS/MS chromatograms showing the m/z transitions measured simultaneously for 50 nM each of MitoGamide-BD, products and deuterated internal standards. Traces are normalized to the maximum total ion count measured during that experiment. **(b)** Typical standards curves for MitoGamide and products prepared in tissue Untreated tissue was spiked with known amounts of MitoGamide-BD, MitoGamide MQA or MitoGamide-BD and prepared in parallel with samples.**Supplementary Fig. 3** Cytokine profiling of plasma samples from 18 weeks old wild-type and Akita mice. Values representing the Akita plasma samples are normalized by the mean value of wild-type samples (*n* = 6–7). Some data points were below the detection limit of the ELISA and for these the detection limit was used in the calculation. **Supplementary Fig. 4** MitoGamide was injected intravenously (100 nmol/mouse, equivalent to 10 mg/kg for mouse weighing 25 g – 30 g) to both wild-type and Akita mice. **(a), (b)** MQA and QA were detected in the spot urine samples from both wild-type and Akita mice at 4 h post i.v. injection. The urine concentrations of MQA and QA are normalized by urine creatinine levels. Values are mean ± SEM (*n* = 5). The differences have been tested by Student’s t test.**Supplementary Fig. 5** No effect of MitoGamide on mitochondrial function in Akita mice. The effect of MitoGamide treatment (10 mg/kg by daily oral gavage for 12 weeks except **b**). **(a)** ATP/ADP ratio in the heart tissues (*n* = 3–7). **(b)** Mitochondrial oxidative stress levels assessed by measuring MitoP/B ratio using LC-MS/MS (*n* = 4–10). 25 nmol MitoB was injected intravenously to mice treated with vehicle or MitoGamide (10 mg/kg) for 1 week. The heart tissues were taken after 3 h of incubation time for the analysis. **(c)** mRNA expression of biomarkers of heart failure in heart tissues (n = 4–6); *Lgals3*, galectin-3; *Nppa*, natriuretic peptide-A; *Nppb*, natriuretic peptide-B; *Gdf15*, growth differentiation factor 15; *Fgf21*, Fibroblast growth factor 21; *Myh7*, myosin heavy chain 7. **(d)** Quantification of mitochondirial density in cardiomyocytes accessed by TEM. **(e)** Tested mtDNA damage level in tissues. mtDNA damage can be detected by a reduced PCR amplication of a long-fragment mtDNA (~10 kbp). Amplication of a long-fragment mtDNA was normalized by amplication of a short fragment (>200 bp) as a control of mtDNA copy number. (n = 5–6). Values are mean ± SEM. Statistical significance has been tested by One-way ANOVA.**Supplementary Fig. 6 (a)** Representative Western blots of autophagy makers. **(b)** Quantification of LC3II expression (normalized to LC3I) and **(c)** p62 and **(d)** Pink 1, both normalized to VDAC in mitochondrial fractions of heart tissues collected after the 12 weeks MitoGamide treatment. (n = 3–6). Values are mean ± SEM and statistical significance has been tested by One-way ANOVA. (PDF 794 kb)
