# Supplementary material for: Confirmation of the Cardioprotective Effect of MitoGamide in the Diabetic Heart
Source: Cardiovasc Drugs Ther. 2020 Sep 26;34(6):823–34. doi: 10.1007/s10557-020-07086-7 (PMC7674384; doi:10.1007/s10557-020-07086-7)
Supplement: Supplementary file 1 — (DOCX 35 kb) [file 10557_2020_7086_MOESM1_ESM.docx]

**Supplementary information**

**Methods**

**Chemical Synthesis**

MitoGamide was synthesised by coupling 3,4-diaminobenzoic acid with (3-aminoprop-1-yl)triphenylphosphonium iodide (prepared as described in (1), using diisopropylcarbodiimide (DIC) in acetonitrile (ACN) so that the product precipitated from solution. Ion exchange and recrystallization gave the methansulfonate salt (Fig S1.). The quinoxalines QA and MQA were prepared by condensing MitoGamide with 40% aqueous glyoxal and methylgloxal, respectively, in ethanol under acid catalysis. MQA was isolated as an inseparable mixture of two regioisomers, A and B. The major regioisomer A was tentatively assigned as 3-methylquinoxaline (MQA1) rather than 2-methylquinoxalines (MQA2), because in water the aldehyde in methylglyoxal is hydrated as the 1,1-diol (2-4). This makes the aldehyde less reactive than the ketone in water and the 3-amino group of MitoGamide is the more electron rich and nucleophilic amino group. The *d_15_* analogues were prepared from (3-aminoprop-1-yl)tri(pentadeuterophenyl)phosphonium iodide by the same method.

*General Conditions.*

All reactions under an inert atmosphere were carried out using oven-dried or flame-dried glassware and solvents were added via syringe. Reagents were obtained from commercial suppliers and used without further purification. Dry solvents were collected from a Puresolv solvent purification system, obtained from commercial suppliers or dried in the laboratory. ^1^H NMR spectra were obtained using Bruker DPX/400 and Bruker-Avance III spectrometers operating at 400 and 500 MHz, ^13^C NMR spectra at 101 and 126 MHz respectively. Signal splitting patterns were described as: singlet (s), doublet (d), triplet (t), quartet (q), multiplet (m), broad singlet (s, broad), or any combination of the above. All coupling constants were recorded in Hz. DEPT was used to assign the signals in ^13^C NMR spectra as C, CH, CH_2_ and CH_3_.

*[3-(3’,4’-Diaminobenzoylamino)propyl]triphenylphosphonium methanesulfonate, (MitoGamide, methanesulfonate salt)*

A solution of (3-aminopropyl)triphenylphosphonium iodide (5.88 g, 13.2 mmol), 3,4-diaminobenzoic acid (2.00 g, 13.1 mmol), and diisopropylcarbodiimide (5.3 mL, 34 mmol) in dry ACN (50 mL) was stirred at RT under Ar for 18 h. The precipitate was collected by filtration and washed with ACN (3 x 25 mL). The residual solvent was removed under high vacuum to give a yellow powder. Ion exchange using Amberlite® IRA-400 charged with mesylate ions followed by recrystallization from methanol gave MitoGamide mesylate as prisms (1.68 g, 54%). Mp: 251-253 °C. δ_H_ (400 MHz, MeOD): 7.90-7.84 (3H, m, Ar-H), 7.81-7.70 (12H, m, Ar-H), 7.19 (1H, d, *J* = 2.1 Hz, H-2’), 7.12 (1H, dd, *J* = 8.2, 2.1 Hz, H-6’), 6.67 (1H, d, *J* = 8.2 Hz, H-5’), 3.51 (2H, t, *J* = 6.5 Hz, NCH_2_), 3.49 – 3.41 (2H, m, PCH_2_), 2.68 (3H, s, CH_3_SO_3_^–^), 2.01 – 1.88 (m, 2H, CH_2_). δ_C_ (101 MHz, MeOD): 170.95 (C), 141.10 (C), 136.32 (d, *J* = 3.1 Hz, CH), 134.76 (d, *J* = 10.1 Hz, CH), 134.75 (C), 131.56 (d, *J* = 12.5 Hz, CH), 124.41 (C), 120.49 (CH), 119.75 (d, *J* = 86.6 Hz, C), 116.41 (CH), 115.53 (CH), 40.48 (d, *J* = 18.5 Hz, CH_2_), 39.53 (CH_3_), 24.01 (d, *J* = 4.0 Hz, CH_2_), 20.69 (d, *J* = 53.2 Hz, CH_2_). δ_P_ (162 MHz: MeOH): 24.01 (s). m/z (HRMS ESI): Found: 454.2029. C_28_H_29_ON_3_P requires *M^+^* (phosphonium cation), 454.2043.

*Triphenyl[3-(quinoxaline-6’-carbonylamino)propyl]phosphonium methanesulfonate (QA, methanesulfonate salt)*

A solution of [3-(3’,4’-diaminobenzoylamino)-prop-1-yl]triphenylphosphonium methanesulfonate (560 mg, 1.02 mmol), acetic acid (1 drop), glyoxal solution 40% in water (150 µl, 1.07 mmol) and ethanol (5 ml) was stirred at RT under Ar for 18 h. After this, the product was extracted with DCM (3 x 20 mL). The combined organics were dried with MgSO_4_, filtered and solvent removed under vacuum to give a brown oil. Column chromatography [SiO_2_, DCM-MeOH (95:5)] followed by ion exchange on Amberlite® IRA-400 charged with mesylate ions gave QA as a brown amorphous solid after solvent removal under vacuum (368 mg, 65%). *R_f_* [SiO_2_, DCM-MeOH (95:5)]: 0.29. δ_H_ (CDCl_3_, 500 MHz): 9.72 (1H, t, *J* = 5.8 Hz, *NH*-CO), 8.89 (1H, d, *J* = 1.9 Hz, H-5’), 8.88 (1H, d, *J* = 1.8 Hz, H-2’ or 3’), 8.85 (1H, d, *J* = 1.8 Hz, H-2’ or 3’), 8.50 (1H, dd, *J* = 8.8 and 2.0 Hz, H-7’), 8.15 (1H, d, *J* = 8.8 Hz, H-8’), 7.76 – 7.71 (9H, m, 6 x *o*-H PPh_3_, 3 $\times$ *p*-H PPh_3_), 7.64-7.60 (6H, m, 6 x *m*-H PPh_3_), 3.84-3.73 (4H, m, P-C*H_2_*, NH-*CH_2_*), 2.86 (3H, s, CH_3_SO_3_^–^)*,* 2.04-1.99 (2H, m, CH_2_*CH_2_*CH_2_). δ_C_ (CDCl_3_, 125 MHz): 166.98 (C), 145.96 (CH), 145.64 (CH), 144.29 (C), 142.71 (C), 135.88 (C), 135.13 (d, *J* = 3.0 Hz, CH), 133.57 (d, *J* = 9.9 Hz, CH), 130.61 (d, *J* = 12.5 Hz, CH), 129.92 (CH), 129.70 (CH), 129.01 (CH), 118.47 (d, *J* = 86.1 Hz, C), 39.79 (CH_3_), 39.65 (d, *J* = 17.4 Hz, CH_2_), 22.54 (d, *J* = 3.9 Hz, CH_2_), 20.16 (d, *J* = 52.6 Hz, CH_2_). δ_P_ (162 MHz: MeOH): 24.46 (s). δ_P_ (162 MHz: MeOH): 24.44 (s). m/z (ESI): Found: 476.1873. C_30_H_27_N_3_OP*^+^* requires *M ^+^* (phosphonium cation), 476.1886.

*[3-(3’-(Methylquinoxaline-6’-carbonylamino)propyl]triphenylphosphonium methanesulfonate (MQA, methanesulfonate salt)*

A solution of [3-(3’,4’-diaminobenzoylamino)-prop-1-yl]triphenylphosphonium mesylate (502 mg, 0.91 mmol), acetic acid (1 drop), methylglyoxal solution 40% in water (165 µl, 1.08 mmol) and ethanol (5 ml) was stirred at RT under Ar for 18 h. After this, the product was extracted with DCM (3 x 20 mL). The combined organics were dried with MgSO_4_, filtered and solvent removed under vacuum to give a brown oil. Column chromatography [SiO_2_, DCM-MeOH (95:5)], ion exchange on Amberlite® IRA-400 resin charged with mesylate ions gave MQA as an 2:1 mixture of regioismers A and B as a brown amorphous solid after solvent was removed under vacuum (188 mg, 36%). *R_f_* [SiO_2_, DCM-MeOH (95:5)]: 0.29. δ_H_ (CDCl_3_, 500 MHz): 9.66 (1H, t, *J* = 5.4 Hz, NH^A+B^), 8.84 (1H, d, *J* = 1.9 Hz, H-5’^A^), 8.79 (1H, d, *J* = 1.8 Hz, H-5’^B^), 8.76 (1H, s, H-2’^A^), 8.73 (1H, s, H-3’^B^), 8.48 (1H, dd, *J* = 8.8, 2.0 Hz, H-7’^A^), 8.45 (1H, dd, *J* = 8.8, 2.0 Hz, H-7’^B^), 8.10 (1H, d, *J* = 8.7 Hz, H-8’^A^), 8.04 (1H, d, *J* = 8.7 Hz, H-8’^B^), 7.76-7.69 (9H, m, Ar-H^A+B^), 7.64-7.59 (6H, m, Ar-H^A+B^), 3.83-3.75 (4H, m, PCH_2_^A+B^ + NCH_2_^A+B^), 2.87 (3H, s, *CH_3_SO_3_^–^*), 2.77 (3H, s, CH_3_^A^), 2.76 (3H, s, CH_3_^B^), 2.04-1.96 (2H, m, CH_2_^A+B^). δ_C_ (CDCl_3_, 125 MHz): 167.10 (C^A^), 167.08 (C^B^), 155.00 (C^A^), 154.51(C^B^), 147.07 (CH^B^), 146.76 (CH^A^), 143.44 (C^A^), 142.29 (C^B^), 141.78 (C^B^), 140.63 (C^A^), 135.72 (C^B^), 135.11 (d, *J* = 3.2 Hz, CH^A+B^), 134.80 (C^A^), 133.57 (d, *J* = 10.0 Hz, CH^A+B^), 130.60 (d, *J* = 12.5 Hz, CH^A+B^), 129.72 (CH^A^), 129.37 (CH^B^), 129.21 (CH^B^), 128.91 (CH^A^), 128.88 (CH^A^), 127.82 (CH^B^), 118.49 (d, *J* = 86.1 Hz, C^A+B^), 39.80 (CH_3_^A+B^), 39.67 (d, *J* = 17.1 Hz, CH_2_^A+B^), 22.86 (CH_3_^A^), 22.73 (CH_3_^B^), 22.57 (d, *J* = 3.2 Hz, CH_2_^A+B^), 20.17 (d, *J* = 52.5 Hz, CH_2_^A+B^). m/z (ESI): Found: 490.2039. C_31_H_29_N_3_OP*^+^* requires *M ^+^* (phosphonium cation), 490.2043.

*[3-(3’,4’-Diaminobenzoylamino)propyl]*-*d_15_*-*triphenylphosphonium methanesulfonate, (d_15_*-*MitoGamide, methanesulfonate salt)*

d_15_-MitoGamide was synthesized on a 0.54 mmol scale in a similar manner to MitoGamide except the final purification was by column chromatography [SiO_2_, DCM-MeOH (95:5)] to give the amide as a brown solid (189 mg, 62%). δ_H_ (400 MHz, MeOD): 7.18 (1H, d, *J* = 2.1 Hz, H-2’), 7.11 (1H, dd, *J* = 8.2, 2.1 Hz, H-6’), 6.67 (1H, d, *J* = 8.2 Hz, H-5’), 3.51 (2H, t, *J* = 6.4 Hz, NCH_2_), 3.50 – 3.42 (2H, m, PCH_2_), 2.69 (3H, s, CH_3_SO_3_^–^), 1.99 – 1.89 (m, 2H, CH_2_). δ_C_ (101 MHz, MeOD): 170.93 (C), 141.06 (C), 135.99-135.60 (m, CD), 134.86 (C), 134.58-134.18 (m, CD), 131.30-130.80 (m, CD), 124.38 (C), 120.45 (CH), 119.52 (d, *J* = 86.5 Hz, C), 116.33 (CH), 115.51 (CH), 40.77 (d, *J* = 18.5 Hz, CH_2_), 39.51 (CH_3_), 23.97 (d, *J* = 3.9 Hz, CH_2_), 20.66 (d, *J* = 53.4 Hz, CH_2_). δ_P_ (162 MHz: MeOH): 23.92 (s). m/z (ESI): Found: 469.2978. C_28_H_14_D_15_ON_3_P requires *M^+^* (phosphonium cation), 469.2984.

*d_15_*-*Triphenyl[3-(quinoxaline-6’-carbonylamino)propyl]phosphonium methanesulfonate (d_15_*-*QA, methanesulfonate salt)*

d_15_-QA was synthesized on a 0.084 mmol scale in a similar manner to QA to give the amide (9 mg, 18%). δ_H_ (CDCl_3_, 500 MHz): 9.79 (1H, t, *J* = 5.8 Hz, *NH*-CO), 8.90 (1H, d, *J* = 1.9 Hz, H-5’), 8.88 (1H, d, *J* = 1.8 Hz, H-2’ or 3’), 8.85 (1H, d, *J* = 1.8 Hz, H-2’ or 3’), 8.56 (1H, dd, *J* = 8.8 and 2.0 Hz, H-7’), 8.16 (1H, d, *J* = 8.8 Hz, H-8’), 3.84-3.76 (4H, m, P-C*H_2_*, NH-*CH_2_*), 2.88 (3H, s, *CH_3_SO_3_^–^*)*,* 2.04-1.96 (2H, m, CH_2_*CH_2_*CH_2_). m/z (ESI): Found: 491.2813. C_30_H_12_D_15_ON_3_P requires *M^+^* (phosphonium cation), 491.2828.

*[3-(3’-(Methylquinoxaline-6’-carbonylamino)propyl]-d_15_-triphenylphosphonium mesylate (d_15_-MQA, methanesulfonate salt).* d_15_-MQA was synthesized on a 0.081 mmol scale in a similar manner to MQA to give the amide, 10 mg (20%), as an 2:1 mixture of regioismers A and B. δ_H_ (CDCl_3_, 500 MHz): 9.84 (1H, t, *J* = 5.8 Hz, NH^A+B^), 8.84 (1H, d, *J* = 2.0 Hz, H-5’^A^), 8.82 (1H, d, *J* = 1.9 Hz, H-5’^B^), 8.77 (1H, s, H-2’^A^), 8.74 (1H, s, H-3’^B^), 8.58 (1H, dd, *J* = 8.8, 2.0 Hz, H-7’^A^), 8.54 (1H, dd, *J* = 8.8, 2.0 Hz, H-7’^B^), 8.12 (1H, d, *J* = 8.7 Hz, H-8’^A^), 8.06 (1H, d, *J* = 8.8 Hz, H-8’^B^), 3.89-3.80 (4H, m, PCH_2_^A+B^ + NCH_2_^A+B^), 2.88 (3H, s, *CH_3_SO_3_^–^*), 2.78 (3H, s, CH_3_^A+B^), 2.01-1.96 (2H, m, CH_2_^A+B^). m/z (ESI): Found: 505.2969. C_31_H_14_D_15_ON_3_P requires *M^+^* (phosphonium cation), 505.2984.

**Quantification of MitoGamide, MQA and QA using LC-MS/MS**

For extraction of MitoGamide and products from tissues, approximately 50 mg sections of tissue were homgenised in 200µL 60%ACN+10mM butanedione for 15 s at 6500rpm in Precellys tubes. Samples were then incubated for 30 min on ice, and then centrifuged for 10 minutes at 14000 xg. Supernatant was transferred to a fresh tube, and pellets were reextracted in 200 µl 60% ACN/0.1% FA, voretexed for 10 s and then centrifuged for 10 minutes at 14000 xg. Supernatants were combined, 10 µL of deuterated internal standard was added and samples were vortexed and left on ice for 30 min. Samples were centrifuged for 10 minutes at 14000 xg, supernatant was filtered and samples were then dried in a speed vac for 2-3 h. Dried samples were resuspended in 250 µl 20% ACN and centrifuged for 10 min at 14000 xg. 200 µL was transferred to an MS vial and samples were stored at -20˚C until analysis. Standard curves were prepared in parallel using untreated tissue spike with known amounts of MitoGamide-BD and products.

Samples were then analysed by LC/MS/MS with multiple reaction monitoring (MRM) in positive ion mode using an I-class Acquity LC attached to a Xevo TQ-S triple quad mass spectrometer (Waters), analysed using MassLynx software. Liquid chromatography was performed at 30ºC using a 2 µL sample injected via flow through needle into an Acquity UPLC BEH C18 1.7 µm, 1x50 mm (Waters, cat no: 186002344). MS buffer A (95 % (v/v) water / 5 % (v/v) acetonitrile / 0.1 % (v/v) formic acid and MS buffer B (90 % (v/v) acetonitrile/ 10 % (v/v) water / 0.1 % (v/v) formic acid) were used at 200 µl/min for the following LC gradient: 0-0.3 min, 5% buffer B; 0.3-3 min, 5%-100% buffer B; 3-4 min, 100 % buffer B, 4.0-4.10, 100 %-5 % buffer B; 4.10-4.60 min, 5% buffer B.

**Mitochondria Isolation**

Rats were killed using the Schedule 1 method of stunning and cervical dislocation in accordance with the United Kingdom Home Office Animals (Scientific Procedures) Act 1986 and the heart rapidly excided. The tissue excised was kept in STE or STEB buffer on ice, until mitochondrial isolation. Equipment used in the procurement and isolation process was detergent-free and pre-rinsed with ice-cold STE or STEB buffer. All steps were carried out at 4 °C.

The procedure was adapted from a previously descripted procedure (2). Heart tissues were washed multiple times in ice-cold STE buffer supplemented with 0.1% (w/V) bovine serum albumin (BSA) to rinse hair and blood and placed in a petri dish. Hearts were cut into quarters, the aorta, blood clots and fat were removed with scissors and then chopped finely with a razor blade. The tissues were washed multiple times with STEB until the buffer was completely clear and homogenised 5 to 8 times using a loose PTFE pestle in a 55 mL Potter-Elvehjem tissue grinder (Wheaton, USA) and then a tight one with roughly 8 strokes. The homogenised tissue was split in two 50 mL plastic centrifuge tube made up to 40 mL circa with STEB and centrifuged (700 xg, 5 min, 4 °C) in a Sorvall RC5C Plus centrifuge with a SS-34 rotor to remove blood and cell debris. The supernatant was decanted through two layers of a pre-wetted muslin into two other centrifuge tubes, the pellet was resuspended, the volume made up to 40 mL circa and both tubes were centrifuged again (700 xg, 5 min, 4 °C) in the same centrifuge. The supernatants were decanted through the two layers of muslin and centrifuged (10,000 xg, 10 min, 4 °C). The mitochondrial pellet was scraped out leaving the blood, combined in a new tube with 40 mL of STEB circa and span again (10,000 xg, 10 min, 4 °C). The supernatant was discarded, and the mitochondrial pellet resuspended in STE without BSA (300 μL circa per heart), kept in ice and used within 4 h in order to assure mitochondria integrity throughout the experiments.

**ATP/ADP assay**

ATP and ADP concentrations were measured using a Luciferase-based assay. Frozen tissue samples were homogenised in ice-cold perchloric acid extractant (3% v/v HClO_4_, 2 mM Na_2_EDTA, 0.5% Triton X-100). Samples diluted to 1 mg/ml, ATP and ADP standards (400 µl) were pH neutralized using a potassium hydroxide solution (2 M KOH, 2 mM Na_2_EDTA, 50 mM MOPS) then centrifuged (17,000 x g for 1 min at 4°C). For ADP measurements, 250 µl of the supernatant was mixed with 250 µl ATP sulfurylase assay buffer (20 mM Na_2_MoO_4_, 5 mM GMP, 0.2 U ATP sulfurylase) then incubated for 30 min at 30°C followed by boiling at 100°C for 5 min to deactivate the enzyme acitivity. Standards (100 µl), samples for ATP (100 µl) or samples for ADP measurement (200 µl) were added to 400 µl Tris-acetate (TA) buffer in luminometer tubes. 10 µl pyruvate kinase solution (100 mM PEP, 6 U pyruvate kinase suspension) were added to one set of samples for ADP measurement and incubated for 30 min at 25°C in the dark to convert ADP to ATP. The other duplicate tube (without addition of pyruvate kinase solution) served as an ATP ‘blank’ value. The samples were then all assayed for ATP content in a Berthold AutoLumat Plus luminometer by addition of 100 µl Luciferase/Luciferin Solution (7.5 mM DTT, 0.4mg/ml BSA, 1.92 µg luciferase/ml, 120 µM D-luciferin). Bioluminescence of the ATP-dependent luciferase activity was measured 3X for 1 min post injection and the data quantified against standard curves.

**Transmission electron microscopy (TEM) image analysis**

Samples were fixed with 2.5% glutaraldehyde in 0.1 M sodium cacodylate buffer pH 7.4 overnight at 4°C, and subsequently incubated with a solution of 1% of tannic acid for 1h. The samples were post-fixed with 1% osmium tetroxide in 0.1M sodium cacodylate buffer for 1 hr at 4°. After three washes with water, samples were dehydrated in a graded ethanol series and embedded in an epoxy resin (Sigma-Aldrich). Ultrathin sections (60-70 nm) were obtained with an Ultrotome V (LKB) ultramicrotome, counterstained with uranyl acetate and lead citrate and viewed with a Tecnai G^2^ (FEI) transmission electron microscope operated at 100 kV. Images were acquired by a Veleta (Olympus Soft Imaging System) digital camera and analysis was performed in a blinded fashion.

**Cell viability assay**

When C2C12 cells reached approximately 50-60% confluency in a 96-well plate, cells were treated with methylglyoxal (1 mM) or glyoxal (1 mM) in 10% FBS DMEM. MitoGamide was co-incubated at final concentrations of 0, 10, 50, 100 µM. After 16 hrs, 10 μl of 5 mg/ml of MTT solution dissolved in the culture medium was given to each well after aspirating the pre-existed medium. After 1.5 hr incubation at 37°C, when the formation of MTT-formazan is observed as blue crystals, the medium was carefully aspirated. MTT-formazan was solublized with 100 μl of DMSO and the intensity was measured colorimetrically at 570 nm using a microplate reader (Spectra Max, Molecular Devices).

**Western blot analysis**

25 μg of tissue lysates in lysis buffer supplemented with protease and phosphatase inhibitor cocktails was mixed with 4 x Laemmli sample buffer and boiled at 95 °C for 5 min. Each sample was loaded into an SDS-polyacrylamide gel and then transferred to a PVDF membrane. Membranes were blocked in 3% BSA in PBST for 30 min and incubated with specific primary antibodies, anti-MG (#MMG-030, Japan Institute for the control of aging) or VDAC (#Ab14734, Abcam) overnight at 4°C. On the following day, membranes were washed three times by TBST and subsequently incubated with appropriate secondary antibodies (#926-68070 or #926-32211, LI-COR) for 1 hr at room temperature. Protein expressions were quantified using Odyssey Imaging system (LI-COR).

**mRNA quantification by qPCR**

RNA was extracted from heart tissues using QIAZOL (#17306, Qiagen) and the RNeasy mini kit (#74104, Qiagen) according to the manufacturer's protocol. 1 μg of isolated RNA was reverse-transcribed to cDNA using SuperScript IV VILO Master Mix (#1176050, ThermoFisher Scientific) according to the manufacturer's protocol without modification. Thereafter, *Taqman* Assays were used to analyze the mRNA expression of *Fgf21* (Mm00840165_g1), *Nppb* (Mm01255770_g1), *Nppa* (Mm0125574_g1), *Gdf15* (Mm00442228_m1), *Lgals3* (Mm00802901_m1), *Myh7* (Mm00600555_m1) and β-actin (Mm02619580_g1) in Applied Biosystem 7500 Real-time PCR system.

**Mitochondrial DNA damage**

QPCR for mitochondrial DNA lesions QPCR for murine mitochondrial DNA lesions was performed using following primers at 10 uM: Forward primer FWD: 5’-GCC AGC CTG ACC CAT AGC CAT AAT-3’. Reverse primer for the long 10090 bp PCR product: REV: 5’-GAG AGA TTT TAT GGG TGT AAT GCG G-3’. Reverse primer for the short 127 bp PCR product: REV: 5’- GCC GGC TGC GTA TTC TAC GTT A -3’. QPCR was performed on 15 ng of sample DNA in 35 µl reactions on a PCR thermocycler (Biometra). 1 U TaKaRa LA Taq per reaction was used with its master mix. Linearity of the PCR reaction was confirmed for each sample by running a 1:2 diluted sample simultaneously. Cycling parameters for the short reaction were 94ºC for 5 minutes followed by 16 cycles of 94ºC for 30s, 64ºC for 45s, 72ºC for 45s, followed by 72ºC for 10 minutes. Conditions for the long amplification were 94ºC for 5 minutes followed by 18 cycles of 94ºC for 15s and 64ºC for 12 minutes, followed by 72ºC for 10 minutes. Concentrations of the PCR product were determined by measuring the emission of the samples at 488 nm and excitation at 525 nm after the addition of picogreen at 1:200 dilution.

**Supplementary References**

(1) Arndt, S. *et al.* Assessment of H_2_S in vivo using the newly developed

mitochondria-targeted mass spectrometry probe MitoA. *J Biol Chem* 2017; **292**: 7761-7773.

(2) Robb, E. L. *et al.* Selective superoxide generation within mitochondria by the targeted redox cycler MitoParaquat. *Free Radic. Biol. Med.*, **2015**, *89*, 883.
