# Supplementary material for: Confirmation of the Cardioprotective Effect of MitoGamide in the Diabetic Heart
Source: Cardiovasc Drugs Ther. 2020 Sep 26;34(6):823–34. doi: 10.1007/s10557-020-07086-7 (PMC7674384; doi:10.1007/s10557-020-07086-7)
Supplement: Supplementary file 2 — Echocardiogrpahy measurements comparing vehicle-treated wild-type, vehicle-treated Akita, and MitoGamide (10 mg/kg given via daily oral gavage for 12 weeks) treated Akita mice at 18 weeks of age. EF; ejection fraction, FS; fractional shortning, CO; cardiac output, SV; stroke volume, LVEDV; left ventricular end-diastolic volume, LVESV; left ventricular end-systolic volume, LVAW; left ventricular anterior wall thickness, LVPW; left ventricular posterior wall thickness, LV mass; left ventricle mass, AoV; peak aortic valve velocity, MV E; mitral valvle E (early) peak velocity; MV A; mitral valve A (atrial contraction) peak velocity, IVRT; isovolumetric relaxation time, IVCT; isovolumetric contraction time, AET; aortic ejection time, E Decel; E peak deceleration, HR; heart rate. Values are mean ± SEM (n = 11–15).Statistical significance has been tested by One-way ANOVA. (PDF 50 kb) [file 10557_2020_7086_MOESM2_ESM.pdf]

Table 1.

Measurement	WT-vehicle	Akita-vehicle	Akita-MitoGamide
EF (%)	53.4±1.3	58.9±1.7	63.1±2.6
FS (%)	27.3±0.8	30.8±1.1	33.8±1.7
CO (ml/min)	19.3±0.9	16.9±0.8	16.7±0.7
SV (µl)	42.2±1.9	37.3±1.5	35.9±1.4
LVV:d (µl)	79.2±1.9	63.7±2.7*	58.3±3.6
LVV:s (µl)	37.0±1.9	26.4±1.8*	22.4±3.0
LVAW:d (mm)	0.8±0.01	0.7±0.03*	0.7±0.03
LVAW:s (mm)	1.0±0.02	1.0±0.05	1.0±0.03
LVPW:d (mm)	0.7±0.03	0.6±0.03*	0.7±0.02
LVPW:s (mm)	0.1±0.04	1.0±0.03	1.0±0.04
LV mass (mg)	117.4±4.6	85.4±4.8*	85.0±3.7
AoV vel (mm/s)	1132±44.4	937.7±31.4*	1006±28.9
MV E vel (mm/s)	775.2±11.3	630.3±36.7*	669.3±16.1
MV A vel (mm/s)	556.7±11.3	552.9±31.1	514.0±17.0
IVRT (ms)	15.5±0.4	20.4±0.9*	19.3±0.7
IVCT (ms)	14.3±0.5	15.2±0.6	14.1±0.6
AET (ms)	52.5±1.2	54.6±0.9	53.9±1.3
E Decel (mm/s^2)	-40680±1738	-29063±2308*	-33129±1216
HR (bpm)	458.6±6.3	452.0±6.6	465.4±7.6
