# Supplementary figures and images for: Confirmation of the Cardioprotective Effect of MitoGamide in the Diabetic Heart
Source: Cardiovasc Drugs Ther. 2020 Sep 26;34(6):823–34. doi: 10.1007/s10557-020-07086-7 (PMC7674384; doi:10.1007/s10557-020-07086-7)

Figure S1.

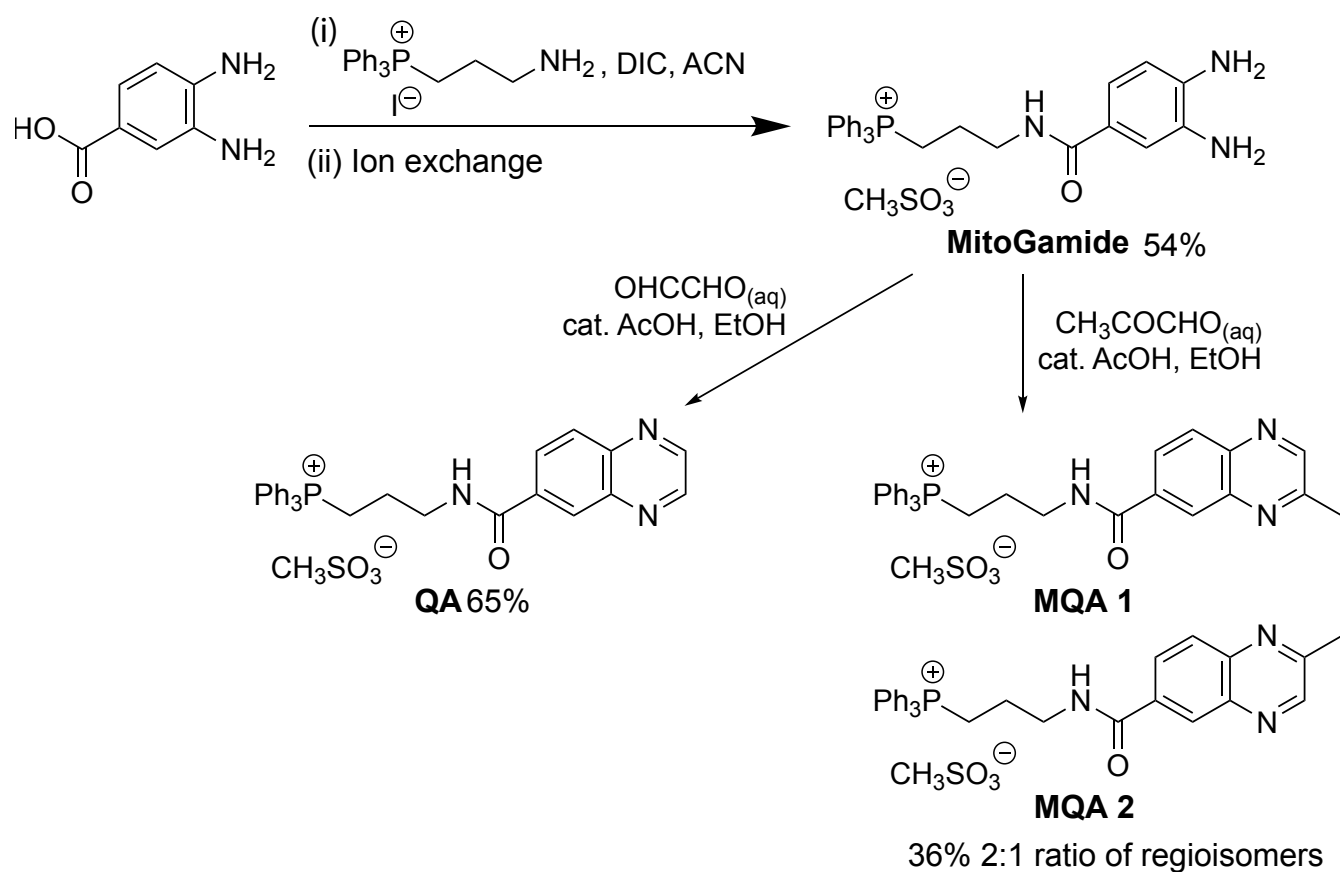

Figure S2.

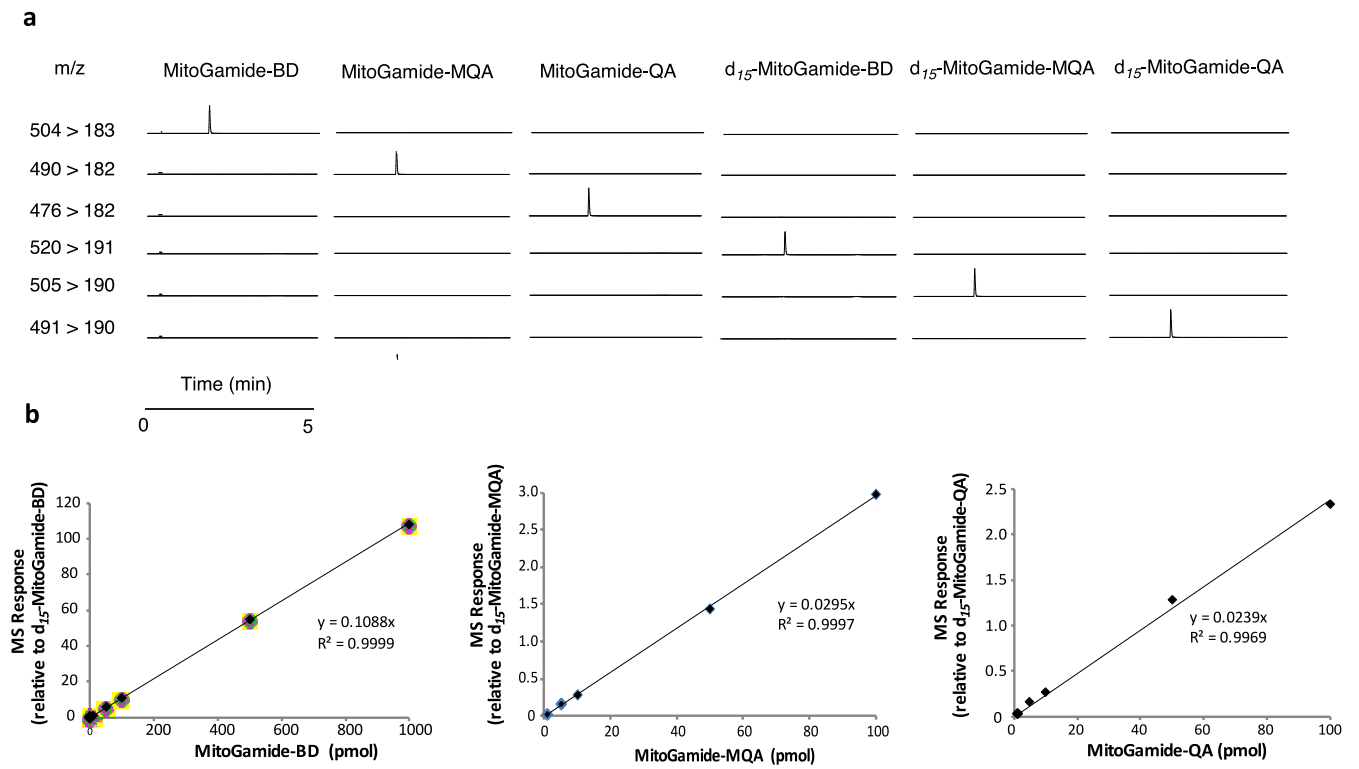

Figure S3.

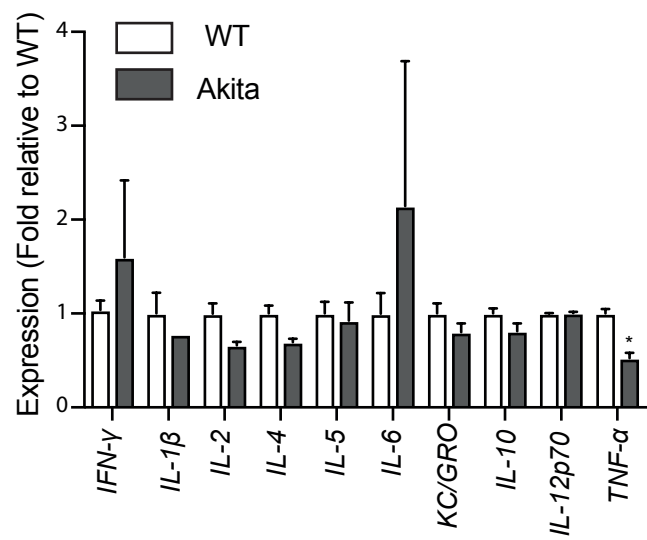

Figure S4.

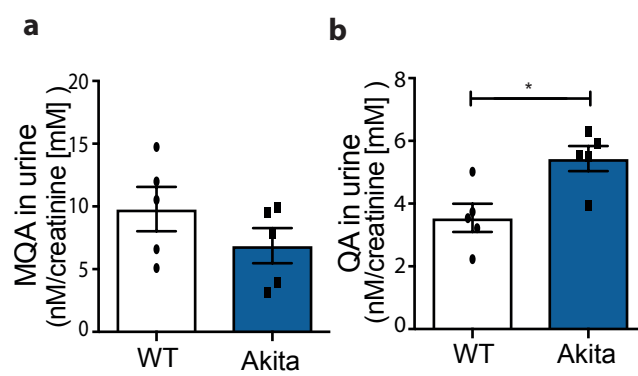

Figure S6.

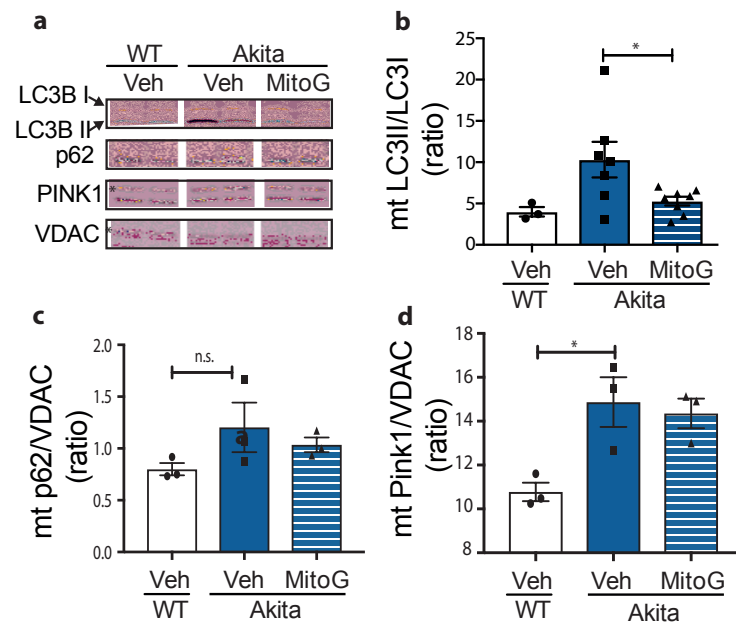

Figure S5.

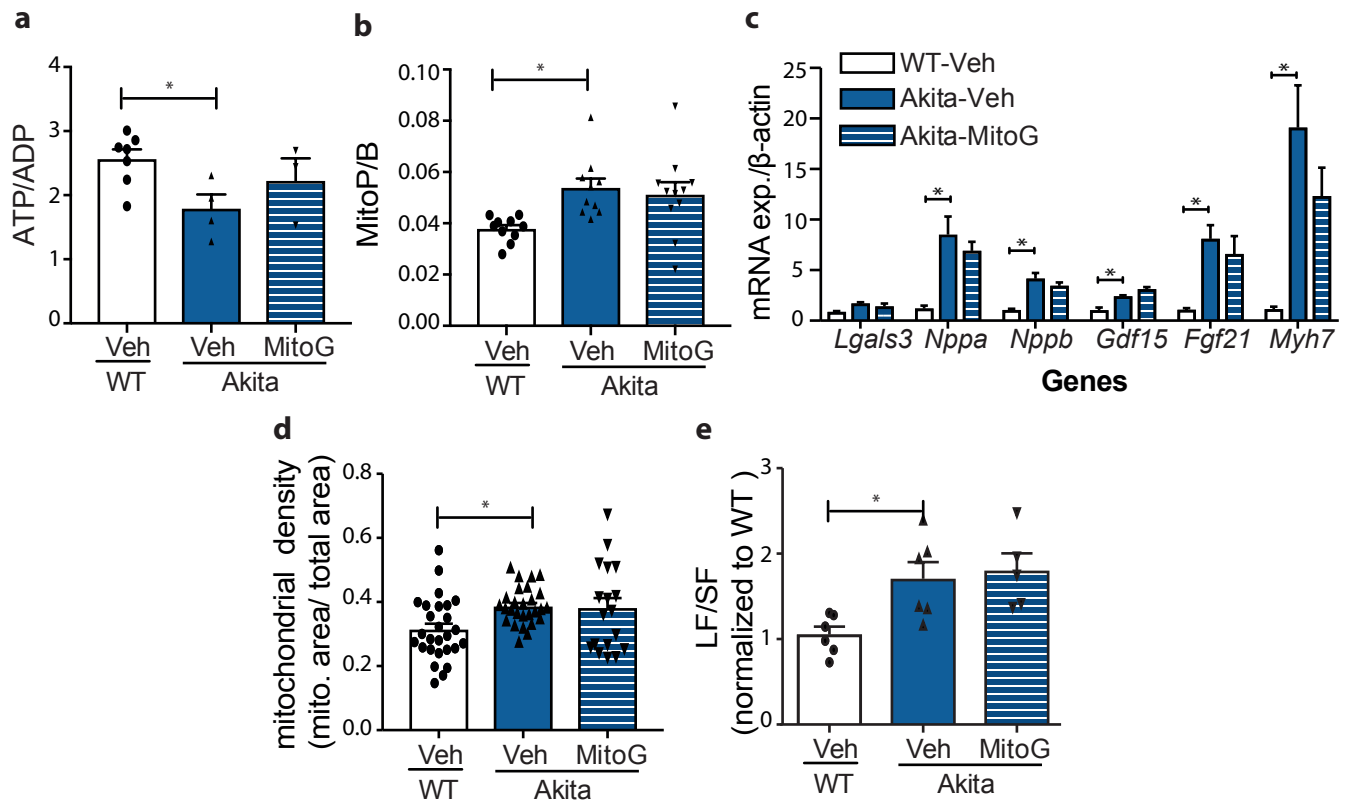

Supplement: Supplementary file 3 — Supplementary Fig. 1 Chemical syntheses of MitoGamide, MQA and QA. Supplementary Fig. 2 LC-MS/MS quantification of MitoGamide and its products. (a) Typical LC-MS/MS chromatograms showing the m/z transitions measured simultaneously for 50 nM each of MitoGamide-BD, products and deuterated internal standards. Traces are normalized to the maximum total ion count measured during that experiment. (b) Typical standards curves for MitoGamide and products prepared in tissue Untreated tissue was spiked with known amounts of MitoGamide-BD, MitoGamide MQA or MitoGamide-BD and prepared in parallel with samples.Supplementary Fig. 3 Cytokine profiling of plasma samples from 18 weeks old wild-type and Akita mice. Values representing the Akita plasma samples are normalized by the mean value of wild-type samples (n = 6–7). Some data points were below the detection limit of the ELISA and for these the detection limit was used in the calculation. Supplementary Fig. 4 MitoGamide was injected intravenously (100 nmol/mouse, equivalent to 10 mg/kg for mouse weighing 25 g – 30 g) to both wild-type and Akita mice. (a), (b) MQA and QA were detected in the spot urine samples from both wild-type and Akita mice at 4 h post i.v. injection. The urine concentrations of MQA and QA are normalized by urine creatinine levels. Values are mean ± SEM (n = 5). The differences have been tested by Student’s t test.Supplementary Fig. 5 No effect of MitoGamide on mitochondrial function in Akita mice. The effect of MitoGamide treatment (10 mg/kg by daily oral gavage for 12 weeks except b). (a) ATP/ADP ratio in the heart tissues (n = 3–7). (b) Mitochondrial oxidative stress levels assessed by measuring MitoP/B ratio using LC-MS/MS (n = 4–10). 25 nmol MitoB was injected intravenously to mice treated with vehicle or MitoGamide (10 mg/kg) for 1 week. The heart tissues were taken after 3 h of incubation time for the analysis. (c) mRNA expression of biomarkers of heart failure in heart tissues (n = 4–6); Lgals3, gale [file 10557_2020_7086_MOESM3_ESM.pdf]
